# Kinematic dataset of actors expressing emotions

**DOI:** 10.1038/s41597-020-00635-7

**Published:** 2020-09-08

**Authors:** Mingming Zhang, Lu Yu, Keye Zhang, Bixuan Du, Bin Zhan, Shaohua Chen, Xiuhao Jiang, Shuai Guo, Jiafeng Zhao, Yang Wang, Bin Wang, Shenglan Liu, Wenbo Luo

**Affiliations:** 1 Research Center of Brain and Cognitive Neuroscience, Liaoning Normal University, Dalian, 116029 Liaoning China; 2 Key Laboratory of Brain and Cognitive Neuroscience, Liaoning Province, Dalian, 116029 Liaoning China; 3 School of Social and Behavioral Sciences, Nanjing University, Nanjing, 210023 Jiangsu China; 4 School of Innovation and Entrepreneurship, Dalian University of Technology, Dalian, 116024 Liaoning China; 5 Faculty of Electronic Information and Electrical Engineering, Dalian University of Technology, Dalian, 116024 Liaoning China

**Keywords:** Human behaviour, Social behaviour, Computer science

## Abstract

Human body movements can convey a variety of emotions and even create advantages in some special life situations. However, how emotion is encoded in body movements has remained unclear. One reason is that there is a lack of public human body kinematic dataset regarding the expressing of various emotions. Therefore, we aimed to produce a comprehensive dataset to assist in recognizing cues from all parts of the body that indicate six basic emotions (happiness, sadness, anger, fear, disgust, surprise) and neutral expression. The present dataset was created using a portable wireless motion capture system. Twenty-two semi-professional actors (half male) completed performances according to the standardized guidance and preferred daily events. A total of 1402 recordings at 125 Hz were collected, consisting of the position and rotation data of 72 anatomical nodes. To our knowledge, this is now the largest emotional kinematic dataset of the human body. We hope this dataset will contribute to multiple fields of research and practice, including social neuroscience, psychiatry, computer vision, and biometric and information forensics.

## Abstract (summary)

**Table:** 

Measurement(s)	body movement coordination trait • emotion/affect behavior trait
Technology Type(s)	motion capture system
Factor Type(s)	emotion category • sex
Sample Characteristic - Organism	Homo sapiens

Machine-accessible metadata file describing the reported data: 10.6084/m9.figshare.12821150

## Background & Summary

Recognizing human emotions is crucial to people’s survival and social communication. There are various carriers and channels of emotional expression. The relevant existing works in both psychology^1^ and computer science^2,3^ mainly focus on human faces and voices as well as emotional scene (context, situations, and conditions). Although the classical theories of emotion have highlighted the significance of body movements since the 19th century^4,5^, as de Gelder^1^ said, “*bodily expressions never occupied centre stage in emotion research*.” In recent decades, psychologists have taken up this issue and found that body movements can provide comparable recognition accuracy relative to facial expression, regardless of static and dynamic conditions^6,7^. Moreover, body movements may show advantages in emotional integration, where multiple emotional stimuli affect each other. For example, when someone is experiencing the most intense emotion of her/his life (e.g., winning the Olympic gold medal), others cannot recognize his/her emotion from just the face and have to use body cues^8^. Similarly, complexities in various situations also exist due to the emotional interactions with voice^9^ and scene^10,11^. Therefore, human body indicators are essential for thorough emotion recognition.

In previous studies, researchers have created some emotional body movement sets using motion capture techniques. These stimulus sets record people’s emotional expression while dancing, walking, and performing other actions^7,12–24^. However, most of these researchers have published finished products (e.g., point-light displays, video clips), but have not included raw kinematic data, and there are fewer recordings in those sets than in those for facial indicators^25^. Some sets consist only of arm movements and do not demonstrate the whole body^26^. Moreover, some studies use only one scenario for a particular emotion (e.g., fear with a pursuing attacker)^6,27^, or the same situational guidance across all emotions (e.g., walking)^14,28^. In daily life, people do not always express their emotions in a single situation or through a single physical posture. Kinematic data includes rich information, such as the position and rotation of body segments, joint angles, and spatio-temporal gait parameters. These points demonstrate the restrictions that have kept researchers from studying how emotion is encoded in body movements and revealing the relationship between quantitative assessment for each joint or body segment and emotion dimensions (e.g., pleasant-unpleasant, valence; deactivated-activated, arousal)^29^. Therefore, there is a lack of public human body kinematic dataset expressing various emotions. Considering this lack of available information, we aimed to produce a comprehensive dataset to assist in recognizing emotional cues from all parts of the body in richer daily situations with more ecological validity.

Taken together, we report a human body kinematic dataset that consists of 1402 trials while expressing six basic emotions (happiness, sadness, anger, fear, disgust, surprise) and neutral. Twenty-two semi-professional actors (half male) participated in this study. A low cost and validated inertial motion capture system with 17 sensors was used^30–32^. The actors performed according to the standardized guide and carefully screened daily events. The resulting kinematic dataset contains the position and rotation data of 72 anatomical nodes, which is stored in the BioVision Hierarchy (BVH) structure. This work expands the scope of emotion recognition and can help us to better understand human’s emotion conveyed via body movements. To the best of our knowledge, this is, at present, the largest emotional kinematic dataset based on the whole human body. These data are expected to be used repeatedly and foster progress in several fields, including social neuroscience, psychiatry, human-computer interaction, computer vision, and biometric and information forensics.

## Methods

### Preparation phase

#### Equipment and environment

**Fig. 1:**
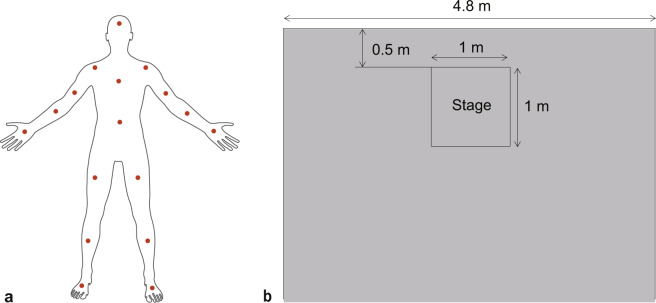
Sensor locations and laboratory environment. (**a**) Seventeen sensors were attached on the actors’ both sides-upper arm and lower arm, hip, spine, head, feet, hands, shoulders, and both legs’ upper leg and lower leg. (**b**) The actors were required to perform in a stage of 1 m × 1 m.

The kinematic data were collected using a wireless motion capture system (Noitom Perception Neuron, Noitom Technology Ltd., Beijing, China) with 17 wearable sensors. This apparatus was connected to the Axis Neuron software (version 3.8.42.8591, Noitom Technology Ltd., Beijing, China) on a laptop computer (Terrans Force T5 SKYLAKE 970 M 67SH1, Windows 10 operating system, Intel Core i7 6700HQ processer). These sensors, with a sampling rate of 125 Hz, were placed on both sides of the actors, including their upper and lower arms, hips, spine, head, feet, hands, shoulders, and both upper and lower legs (see Fig. 1a). The tasks were conducted in a quiet laboratory (see Fig. 1b). The actors needed to execute each performance in a square stage of 1 m × 1 m that was 0.5 m from a wall. Proper and limited performance space would also control the horizontal distance between the actor and the object of emotions so that the horizontal displacement of all recordings was not very different.

#### Scenarios

In order to guide the actors to perform in a typical and natural manner, we created 70 daily event scenarios (10 for each emotion and neutral; see Supplementary File 1) according to the basic concepts of emotions^33–35^ and previous research^6,7,20–22,26,27,36^. To test the validity of the scenarios, 70 college students (mean age = 23.10 years, SD = 1.64, 42 females) were required to classify the emotion expressed in the scenarios displayed randomly on a seven-alternative forced-choice questionnaire (see Supplementary File 2). The five most recognizable scenarios for each emotion were retained (see Online-only Table 1). Note that, for emotional but not neutral recordings, free (non-scripted) performances were added, during which the performance was spontaneous, and the actors were free to interpret and express the emotions as they thought fit, not be restricted by the scenarios. Thus, there were 35 scenarios used in the recording phase.

#### Actors

Another group of 24 college students (mean age = 20.75 years, SD = 1.92; mean height = 1.69 m, SD = 0.07; mean weight = 58.52 kg, SD = 12.42; mean BMI = 20.25, SD = 3.30) was recruited from the drama and dance clubs of the Dalian University of Technology to perform as actors for this study (see Table 1). Actors F04 and F13 were excluded because they dropped out. All actors were physically and mentally healthy and right-handed. Each actor gave their written informed consent before performing and was told that their motion data were to be used only for scientific research. The study was approved by the Human Research Institutional Review Board of Liaoning Normal University in accordance with the Declaration of Helsinki (1991). After the recording phase, the actors were paid appropriately.

**Table 1:** Demographic information of the actors.

Actor ID	Gender	Age (years)	Height (m)	Weight (kg)	BMI	Dominant Hand	Note
F01	Female	20	1.66	52.00	18.87	Right	
F02	Female	20	1.65	62.00	22.77	Right	
F03	Female	23	1.67	55.00	19.72	Right	
F04	Female	24	1.62	53.00	20.20	Right	Drop out
F05	Female	24	1.65	49.00	18.00	Right	
F06	Female	19	1.60	47.00	18.36	Right	
F07	Female	19	1.72	58.00	19.61	Right	
F08	Female	21	1.58	49.00	19.63	Right	
F09	Female	21	1.72	48.00	16.22	Right	
F10	Female	20	1.70	48.00	16.61	Right	
F11	Female	19	1.58	46.00	18.43	Right	
F12	Female	21	1.65	54.00	19.83	Right	
F13	Female	20	1.60	44.00	17.19	Right	Drop out
M01	Male	25	1.75	65.00	21.22	Right	
M02	Male	19	1.70	51.00	17.65	Right	
M03	Male	21	1.72	67.00	22.65	Right	
M04	Male	21	1.73	90.00	30.07	Right	
M05	Male	24	1.75	68.00	22.20	Right	
M06	Male	20	1.80	67.00	20.68	Right	
M07	Male	18	1.72	57.10	19.30	Right	
M08	Male	19	1.80	92.50	28.55	Right	
M09	Male	19	1.75	57.00	18.61	Right	
M10	Male	21	1.80	60.00	18.52	Right	
M11	Male	20	1.75	65.00	21.22	Right	

### Recording phase

**Fig. 2:**
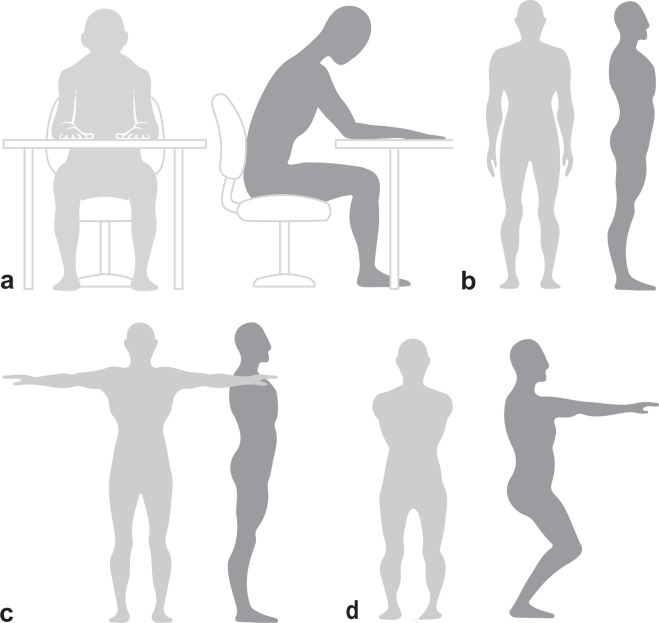
Four-step calibration procedure. (**a**) Steady pose. (**b**) A-pose. (**c**) T-pose. (**d**) S-pose. The figures are taken from another source with permission (https://neuronmocap.com/content/axis-neuron).

The actors wore black tights, and 17 Neuron sensors were attached to the corresponding body location. A four-step calibration procedure using four successive static poses was done for the Axis Neuron software before performances and when necessary (e.g., bad WIFI signal or after a rest) (see Fig. 2; for details, see https://neuronmocap.com/content/axis-neuron). To prevent inconsistencies in interpretation by different actors, standardized instructions were given before each recording (see Supplementary File 3). The actors started in a neutral stance (i.e., facing forward and arms naturally at sides). For each kind of emotion, we successively asked the actors to give a six-second free performance based on their self-understanding and the scenario performance. The order of emotions displayed for the actors was random, and the order of scenarios within each emotion was random as well. When the actors were ready and we said “start”, the Axis Neuron software simultaneously recorded the motion data. After each performance, we reviewed it and evaluated the signal quality; hence, some performances needed to be repeated several times. The recording phase took approximately two hours, during which the actors could have a rest when they felt tired.

## Data Records

A total of 1406 trials were collected. The commercial Axis Neuron software uses a RAW file format to store data. We exported those RAW trial files to BVH files, as it is a standard file format that can be analyzed using various software (e.g., 3ds Max, https://www.autodesk.com/products/3ds-max; MotionBuilder, https://www.autodesk.com/products/motionbuilder). Four raw files were impaired during this process (i.e., F09D0V1, M04F4V2, M06N1V1, and M06SU1V1). Therefore, the human body kinematic dataset—available from 10.13026/kg8b-1t49^37^ (mean duration = 7.22 s, SD = 1.57)—that was created consists of 1402 trials expressing six emotions and neutral.

Each actor has his/her own folder, named as the actor ID (see Table 1), consisting of BVH files for all emotions. Each trial was named systematically as “<actor_ID><emotion><scenario_ID><version>” (for details, see file_info.csv in 10.13026/kg8b-1t49^37^), where “actor_ID” represents the actor ID; “emotion” includes happiness (H), sadness (SA), neutral (N), anger (A), disgust (D), fear (F), and surprise (SU); “scenario_ID” consists of the free (indicated as 0) and scenario performance indicated with the corresponding numeral from 1 to 5; and “version” denotes the number of repetitions.

**Fig. 3:**
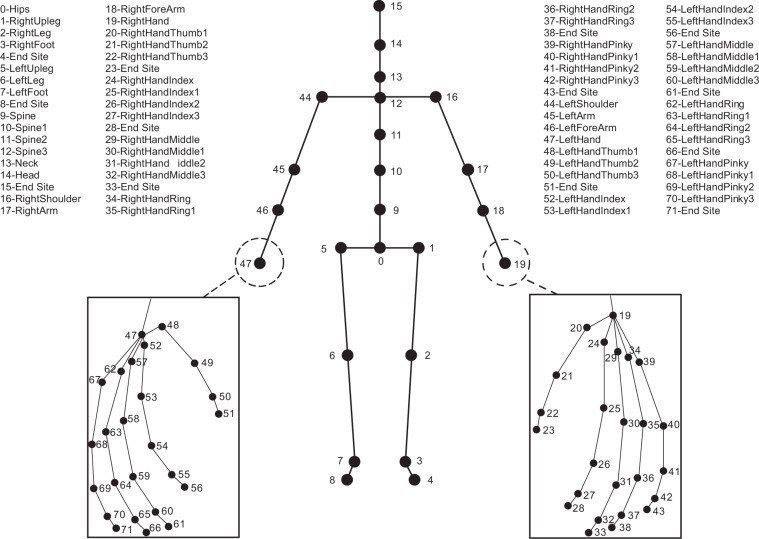
BVH skeletal structure. The approximate anatomical position and corresponding description of 72 nodes are given. Details of the hands are shown in the rectangles.

A BVH file contains ASCII text and two sections (i.e., HIERARCHY and MOTION). Beginning with the keyword HIERARCHY, this section defines the joint tree, the name of each node, the number of channels, and the relative position between joints (i.e., the bone length of each part of the human body). There are totally 72 nodes data (i.e., 1 Root, 58 Joints, and 13 End Sites) in this section (see Fig. 3), which are calculated by the commercial Axis Neuron software according to the 17 sensors (see Fig. 1a). The MOTION section records the motion data. According to the joint sequence defined, the data of each frame is provided, and the position and rotation information of each joint node is recorded. There are some legends in a BVH file:

- HIERARCHY: beginning of the header section
- ROOT: location of the Hips (see Fig. 3)
- JOINT: location of the skeletal joint refers to the parent-joint (see Fig. 3)
- CHANNELS: number of channels including position and rotation channels
- OFFSET: X, Y, and Z offsets of the segment relative to its parent-joint
- End Site: end of a JOINT which has no child-joint (see Fig. 3)
- MOTION: beginning of the second section
- FRAMES: numbers of frames
- Frame Time: sampling time per frame

## Technical Validation

### Scenario

To improve the reality and recognition of actors’ performances, we conducted a pilot study (see Methods) and selected the top five recognizable daily events that had the highest accuracy for each emotion (mean ± SD, happiness: 0.994 ± 0.013; anger: 0.917 ± 0.040; sadness: 0.957 ± 0.026; fear: 0.971 ± 0.023; disgust: 0.906 ± 0.041; surprise: 0.871 ± 0.030; neutral: 0.920 ± 0.042).

### Calibration

**Fig. 4:**
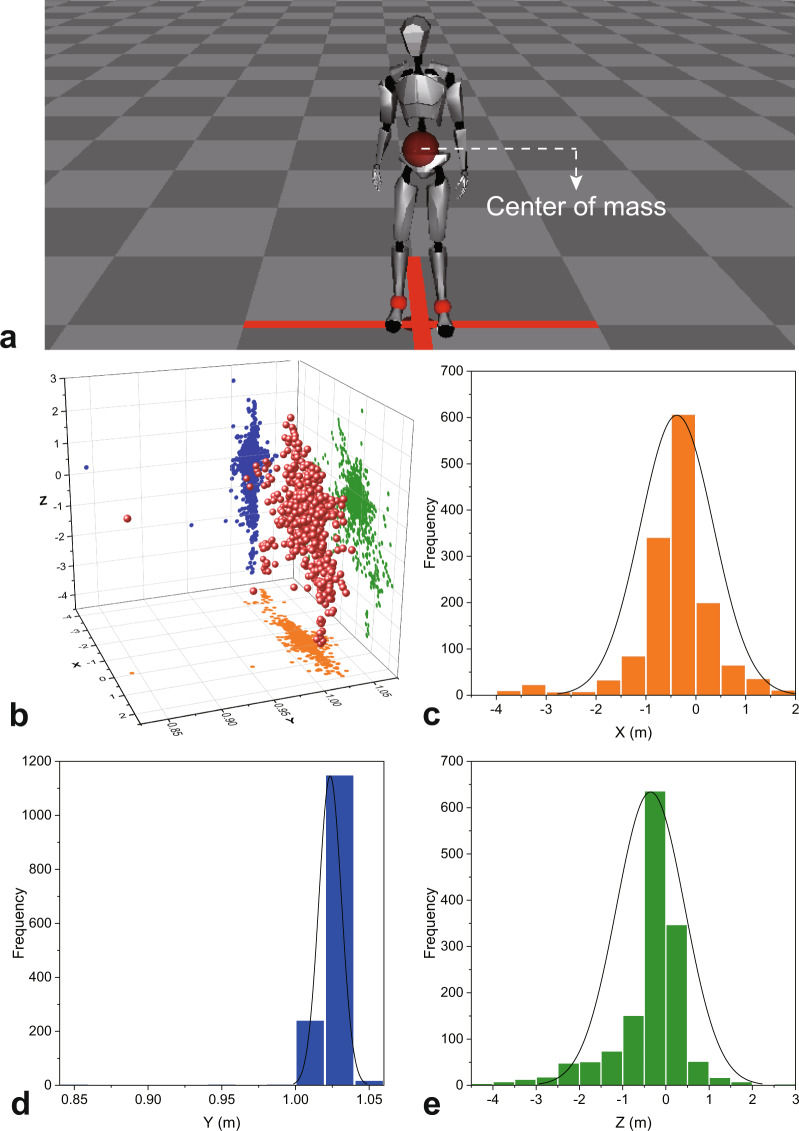
The location of mass center and three-dimensional distribution of the mass center of the first frame. (**a**) A big red transparent ball represents the mass center of the model in the recording software (this figure is taken from another source with permission, https://neuronmocap.com/content/axis-neuron). (**b**) 3D scatter of the mass center of the first frame. (**c**) The distribution of X position. (**d**) The distribution of Y position. (**e**) The distribution of Z position. Figure 4 shows that, after the calibration procedure, the initial spatial position of the actors across all performances are relatively centralized and consistent, reflecting a good calibration quality in the present study.

As described in the recording phase (see Methods), the motion capture system was calibrated with the four-step calibration procedure before performance and as needed. We also reviewed and visually checked the quality of the motion signal and the naturalness of performances trial by trial. After all sensors have been calibrated, the pose of the model in the recording software will be consistent with the actor’s initial stance (i.e., facing forward and arms naturally at sides; see Fig. 4a), and the spatial position of the mass center across all models in the recording software will be relatively stable. Otherwise, the model will be deformed, and the initial spatial position of the mass center will be inconsistent across all performances. Therefore, the spatial positions of the first frame mass center across all recordings can reflect the calibration quality. To evaluate the calibration quality, we used the Axis Neuron software and extracted the X, Y, and Z positions of the mass center (see Fig. 4a) of the first frame from each recording (see https://physionet.org/content/kinematic-actors-emotions/2.1.0/). The data distribution in these three dimensions was relatively centralized, showing that the initial states of the actors would be consistent (see Fig. 4b–e), which suggests a good calibration quality in our study.

## Usage Notes

BVH files can be imported directly into 3ds Max (https://www.autodesk.com/products/3ds-max), MotionBuilder (https://www.autodesk.com/products/motionbuilder), and other 3D applications. Therefore, these data can be used to build different avatars in virtual reality and augmented reality products. Previous studies on emotion recognition in the field of computer and information science have mainly focused on human faces and voices; hence, the dataset created in this study can improve current technologies and contribution to scientific advancements. In the fields of psychiatry and psychology, researchers can also create experimental stimuli based on the present study, such as emotional point-light displays that contain biological motion information^38^. Such material has been applied in the field of social cognitive impairment and can contribute to the clinical diagnosis of autistic spectrum disorders, schizophrenia, and other conditions of psychiatric patients^39,40^.

Amongst the trials produced, some special trials should be noted. Although we asked the actors to complete each performance within six seconds, some trials are, in fact, much shorter or longer because of the difference in human time perception or operator error (e.g., 21.84 s for F07SA0V1, 2.688 s for M01D1V2). Future studies aim to be more consistent by avoiding these trial discrepancies when possible.

Although we have got richer data, reflecting the variability of body movements by creating more daily scenarios and recruiting more actors, and the present set also gives researchers more opportunities to select experimental materials, the differences among the scenarios in the same emotion may bring some “noise”. For example, if the object of anger is a proximal dog, the expression may be targeted at a lower vertical level than if the object is a car speeding off. We will further examine the specific relationship between scenario-induced movement and subjective emotional experience (e.g., emotional intensity, valence, and arousal).

Because all the actors were told that their motion data were to be used only for scientific research before the performance, we followed the recommendation of PhysioNet and chose a suitable license as data use agreement (Restricted Health Data License 1.5.0,

https://physionet.org/content/kinematic-actors-emotions/view-license/2.1.0/). Therefore, users need to sign this agreement online before downloading and using the present dataset.

## Supplementary information

Supplementary File 1

Supplementary File 2

Supplementary File 3

## Data Availability

The Matlab code used to extract the frame number of BVH files to calculate durations can be found at https://physionet.org/content/kinematic-actors-emotions/2.1.0/.

## Online-only Table

**Online-only Table 1:** The five most recognizable scenarios for each emotion used in the recording phase.

Scenario ID	Scenario	Recognition accuracy (%)
Happiness		
H1	Zhang is admitted to his/her favorite university.	100
H2	Zhang receives a notice from the boss that the salary will be raised from next month.	100
H3	Zhang spends half a month on a plan, and it is highly approved by the bosses.	100
H4	Zhang is about to travel around the world soon.	100
H5	Zhang’s favorite basketball team wins the NBA championship.	97.1
Anger		
A1	Zhang keeps awake because of the noise from the neighbor at 3 o’clock a.m.	97.1
A2	Zhang’s dog bites the leather sofa in the living room.	92.9
A3	Zhang’s bike seat is stolen.	92.9
A4	Zhang is splashed with water by the speeding car.	88.6
A5	Zhang does the same job as the colleague but only gets half the salary.	87.1
Sadness		
SA1	Zhang learns that his/her best friend has leukemia.	98.6
SA2	Zhang’s father dies in a car accident.	97.1
SA3	Zhang’s score is only 3 points short of his/her favorite university.	97.1
SA4	Zhang fails to pass the company’s year-end review, so he/she can’t get promoted.	92.9
SA5	Someone Zhang has a secret crush on politely refuses him/her.	92.9
Fear		
F1	A man with a kitchen knife is cutting towards Zhang.	100
F2	Zhang is surrounded by a pack of wolves.	98.6
F3	Zhang accidentally breaks an antique vase in the boss’s office.	97.1
F4	A runaway car rushes towards Zhang.	95.7
F5	A strong earthquake happens in Zhang’s town.	94.3
Disgust		
D1	Zhang sees a large pool of yellow vomit on the ground in front of him/her.	94.3
D2	While riding a bicycle, a fly flies into Zhang’s mouth.	92.9
D3	Zhang accidentally gets a hand of excrement when using the toilet.	92.9
D4	The garbage can in Zhang’s community emits a stench.	88.6
D5	The man standing by Zhang has a body odor on the bus in the morning rush.	84.3
Surprise		
SU1	Zhang sees his/her shy colleague playing rock and roll on the stage.	90.0
SU2	Zhang reads the news that the world’s youngest mother is only five years old.	85.7
SU3	In the cold winter, Zhang sees a man wearing short-sleeved shorts on the street.	90.0
SU4	Zhang finds that the ugly girl ten years ago has become a beautiful woman.	87.1
SU5	Zhang sees a pig breaking into the classroom.	82.9
Neutral		
N1	Zhang picks up the glass and drinks water.	95.7
N2	Zhang is taking the key to open the door.	92.9
N3	Zhang taps on both sides of the thighs.	91.4
N4	Zhang squats and stands up.	91.4
N5	Zhang is marking time.	88.6
