# Supplementary material for: Kinematic dataset of actors expressing emotions
Source: Sci Data. 2020 Sep 8;7:292. doi: 10.1038/s41597-020-00635-7 (PMC7478954; doi:10.1038/s41597-020-00635-7)
Supplement: Supplementary file 1 [file 41597_2020_635_MOESM1_ESM.docx]

| **Questionnaire ID** | **Scenario** | **Emotion** |
| --- | --- | --- |
| 11 | Zhang is broken up by the boyfriend/girlfriend. | Sadness |
| 12 | Zhang's father dies in a car accident. | Sadness |
| 14 | Zhang's favorite football team is knocked out in the semifinal. | Sadness |
| 50 | Zhang fails to pass the company's year-end review, so he/she can’t get promoted. | Sadness |
| 52 | Zhang learns that his/her best friend has leukemia. | Sadness |
| 56 | Zhang drops the new iPhone into the water accidentally. | Sadness |
| 58 | Zhang is fired because of his/her gross negligence. | Sadness |
| 61 | Someone Zhang has a secret crush on politely refuses him/her. | Sadness |
| 63 | Zhang is saying goodbye to boyfriend/girlfriend, and they will start exotic love about two years. | Sadness |
| 66 | Zhang's score is only 3 points short of his/her favorite university. | Sadness |
| 15 | Zhang's dog bites the leather sofa in the living room. | Anger |
| 18 | Zhang keeps awake because of the noise from the neighbor at 3 o'clock a.m. | Anger |
| 25 | Zhang is quarreling with his/her friend. | Anger |
| 36 | Zhang finds the boyfriend/girlfriend is cheating on him/her. | Anger |
| 41 | Zhang's travel plan is upset by others for no reason. | Anger |
| 42 | While watching a movie in the cinema, two people beside Zhang are talking all the time. | Anger |
| 47 | Zhang makes an appointment with the colleague at noon, but the colleague arrives two hours late. | Anger |
| 55 | Zhang's bike seat is stolen. | Anger |
| 62 | Zhang is splashed with water by the speeding car. | Anger |
| 65 | Zhang does the same job as the colleague but only gets half the salary. | Anger |
| 3 | Zhang is listening to his/her favorite singer's concert. | Happiness |
| 6 | Zhang meets the best friend in the street whom he/she hasn’t seen for a long time. | Happiness |
| 8 | Zhang's best friend is getting married today. | Happiness |
| 13 | Zhang's favorite basketball team wins the NBA championship. | Happiness |
| 17 | Zhang is admitted to his/her favorite university. | Happiness |
| 26 | Zhang receives a notice from the boss that the salary will be raised from next month. | Happiness |
| 32 | Zhang spends half a month on a plan, and it is highly approved by the bosses. | Happiness |
| 45 | Zhang is watching a comedy show. | Happiness |
| 59 | Zhang is about to travel around the world soon. | Happiness |
| 70 | The person that Zhang loves secretly confesses his/her love to Zhang actively. | Happiness |
| 10 | A runaway car rushes towards Zhang. | Fear |
| 16 | A man with a kitchen knife is cutting towards Zhang. | Fear |
| 19 | Zhang accidentally breaks an antique vase in the boss's office. | Fear |
| 23 | Zhang sees a group of people with knives are looting in front of him. | Fear |
| 24 | A strong earthquake happens in Zhang's town. | Fear |
| 29 | A snake suddenly appears when Zhang is walking. | Fear |
| 38 | Zhang is about to undergo a craniotomy. | Fear |
| 39 | A robber with pistol asks Zhang to open him/her wallet. | Fear |
| 46 | The police catch Zhang stealing. | Fear |
| 51 | Zhang is surrounded by a pack of wolves. | Fear |
| 1 | Zhang sees a large pool of yellow vomit on the ground in front of him/her. | Disgust |
| 2 | Zhang's friend is talking with the garlic smell. | Disgust |
| 4 | The man standing by Zhang has a body odor on the bus in the morning rush. | Disgust |
| 27 | While riding a bicycle, a fly flies into Zhang's mouth. | Disgust |
| 31 | Zhang accidentally gets a hand of excrement when using the toilet. | Disgust |
| 34 | Zhang is holding a pair of dirty underpants. | Disgust |
| 43 | Zhang sees that the colleague's hair is covered with dandruff, greasy and dirty. | Disgust |
| 48 | Zhang takes the bread from the refrigerator and finds it moldy and covered with mould. | Disgust |
| 54 | The garbage can in Zhang's community emits a stench. | Disgust |
| 57 | Zhang bites the apple and finds there is still half a worm in it. | Disgust |
| 7 | When Zhang comes into the office, he/she finds the usual decent leader is watching a porn. | Surprise |
| 9 | On the way, a stranger suddenly reaches out his/her hand and gives Zhang a paintbrush. | Surprise |
| 28 | Zhang sees his/her shy colleague playing rock and roll on the stage. | Surprise |
| 30 | Zhang reads the news that the world's youngest mother is only five years old. | Surprise |
| 37 | In the cold winter, Zhang sees a man wearing short-sleeved shorts on the street. | Surprise |
| 40 | Zhang is watching a performance of a car flying over the Yellow River. | Surprise |
| 49 | Zhang is standing on the roadside when suddenly a large group of children runs past him /her. | Surprise |
| 64 | Zhang finds that the ugly girl ten years ago has become a beautiful woman. | Surprise |
| 67 | Zhang sees a pig breaking into the classroom. | Surprise |
| 68 | Zhang is listening to a lecture when he/she sees the teacher suddenly jumps onto the platform. | Surprise |
| 5 | Zhang pats the dust on the sleeve. | Neutral |
| 20 | Zhang is spinning. | Neutral |
| 21 | Zhang taps on both sides of the thighs. | Neutral |
| 22 | Zhang is sweeping the floor. | Neutral |
| 33 | Zhang is doing chest expansion exercises. | Neutral |
| 35 | Zhang is marking time. | Neutral |
| 44 | Zhang squats and stands up. | Neutral |
| 53 | Zhang tidies up the collar when he/she gets dressed. | Neutral |
| 60 | Zhang is taking the key to open the door. | Neutral |
| 69 | Zhang picks up the glass and drinks water. | Neutral |
