# Supplementary material for: Kinematic dataset of actors expressing emotions
Source: Sci Data. 2020 Sep 8;7:292. doi: 10.1038/s41597-020-00635-7 (PMC7478954; doi:10.1038/s41597-020-00635-7)
Supplement: Supplementary file 2 [file 41597_2020_635_MOESM2_ESM.docx]

**【Introduction】**

Please read each sentence carefully, imagine how you would feel **if you were Zhang**, and tick the corresponding emotional words at the end of the sentence. There are sixty sentences! Among them, 1= happiness, 2= anger, 3= sadness, 4= fear, 5= disgust, and 6= neutral (neutral means that it does not contain any emotion).

**Note: please choose according to your first feeling. Just answer truthfully. So please give the choice that best fits you. Please do not make multi-choice or omit!**

1. Age:_______ years old
2. Sex: (1) male (2) female
3. Major:___________________

背面还有哦！

| **Number** | **Scenes** | **Happiness** | **Anger** | **Sadness** | **Fear** | **Disgust** | **Surprise** | **Neutral** |
| --- | --- | --- | --- | --- | --- | --- | --- | --- |
|  | Zhang sees a large pool of yellow vomit on the ground in front of him/her. | 1 | 2 | 3 | 4 | 5 | 6 | 7 |
|  | Zhang's friend is talking with the garlic smell. | 1 | 2 | 3 | 4 | 5 | 6 | 7 |
|  | Zhang is listening to his/her favorite singer's concert. | 1 | 2 | 3 | 4 | 5 | 6 | 7 |
|  | The man standing by Zhang has a body odor on the bus in the morning rush. | 1 | 2 | 3 | 4 | 5 | 6 | 7 |
|  | Zhang pats the dust on the sleeve. | 1 | 2 | 3 | 4 | 5 | 6 | 7 |
|  | Zhang meets the best friend in the street whom he/she hasn’t seen for a long time. | 1 | 2 | 3 | 4 | 5 | 6 | 7 |
|  | When Zhang comes into the office, he/she finds the usual decent leader is watching a porn. | 1 | 2 | 3 | 4 | 5 | 6 | 7 |
|  | Zhang's best friend is getting married today. | 1 | 2 | 3 | 4 | 5 | 6 | 7 |
|  | On the way, a stranger suddenly reaches out his/her hand and gives Zhang a paintbrush. | 1 | 2 | 3 | 4 | 5 | 6 | 7 |
|  | A runaway car rushes towards Zhang. | 1 | 2 | 3 | 4 | 5 | 6 | 7 |
|  | Zhang is broken up by the boyfriend/girlfriend. | 1 | 2 | 3 | 4 | 5 | 6 | 7 |
|  | Zhang's father dies in a car accident. | 1 | 2 | 3 | 4 | 5 | 6 | 7 |
|  | Zhang's favorite basketball team wins the NBA championship. | 1 | 2 | 3 | 4 | 5 | 6 | 7 |
|  | Zhang's favorite football team is knocked out in the semifinal. | 1 | 2 | 3 | 4 | 5 | 6 | 7 |
|  | Zhang's dog bites the leather sofa in the living room. | 1 | 2 | 3 | 4 | 5 | 6 | 7 |
|  | A man with a kitchen knife is cutting towards Zhang. | 1 | 2 | 3 | 4 | 5 | 6 | 7 |
|  | Zhang is admitted to his/her favorite university. | 1 | 2 | 3 | 4 | 5 | 6 | 7 |
|  | Zhang keeps awake because of the noise from the neighbor at 3 o'clock a.m. | 1 | 2 | 3 | 4 | 5 | 6 | 7 |
|  | Zhang accidentally breaks an antique vase in the boss's office. | 1 | 2 | 3 | 4 | 5 | 6 | 7 |
|  | Zhang is spinning. | 1 | 2 | 3 | 4 | 5 | 6 | 7 |
|  | Zhang taps on both sides of the thighs. | 1 | 2 | 3 | 4 | 5 | 6 | 7 |
|  | Zhang is sweeping the floor. | 1 | 2 | 3 | 4 | 5 | 6 | 7 |
|  | Zhang sees a group of people with knives are looting in front of him. | 1 | 2 | 3 | 4 | 5 | 6 | 7 |
|  | A strong earthquake happens in Zhang's town. | 1 | 2 | 3 | 4 | 5 | 6 | 7 |
|  | Zhang is quarreling with his/her friend. | 1 | 2 | 3 | 4 | 5 | 6 | 7 |
|  | Zhang receives a notice from the boss that the salary will be raised from next month. | 1 | 2 | 3 | 4 | 5 | 6 | 7 |
|  | While riding a bicycle, a fly flies into Zhang's mouth. | 1 | 2 | 3 | 4 | 5 | 6 | 7 |
|  | Zhang sees his/her shy colleague playing rock and roll on the stage. | 1 | 2 | 3 | 4 | 5 | 6 | 7 |
|  | A snake suddenly appears when Zhang is walking. | 1 | 2 | 3 | 4 | 5 | 6 | 7 |
|  | Zhang reads the news that the world's youngest mother is only five years old. | 1 | 2 | 3 | 4 | 5 | 6 | 7 |
|  | Zhang accidentally gets a hand of excrement when using the toilet. | 1 | 2 | 3 | 4 | 5 | 6 | 7 |
|  | Zhang spends half a month on a plan, and it is highly approved by the bosses. | 1 | 2 | 3 | 4 | 5 | 6 | 7 |
|  | Zhang is doing chest expansion exercises. | 1 | 2 | 3 | 4 | 5 | 6 | 7 |
|  | Zhang is holding a pair of dirty underpants. | 1 | 2 | 3 | 4 | 5 | 6 | 7 |
|  | Zhang is marking time. | 1 | 2 | 3 | 4 | 5 | 6 | 7 |
|  | Zhang finds the boyfriend/girlfriend is cheating on him/her. | 1 | 2 | 3 | 4 | 5 | 6 | 7 |
|  | In the cold winter, Zhang sees a man wearing short-sleeved shorts on the street. | 1 | 2 | 3 | 4 | 5 | 6 | 7 |
|  | Zhang is about to undergo a craniotomy. | 1 | 2 | 3 | 4 | 5 | 6 | 7 |
|  | A robber with pistol asks Zhang to open him/her wallet. | 1 | 2 | 3 | 4 | 5 | 6 | 7 |
|  | Zhang is watching a performance of a car flying over the Yellow River. | 1 | 2 | 3 | 4 | 5 | 6 | 7 |
|  | Zhang's travel plan is upset by others for no reason. | 1 | 2 | 3 | 4 | 5 | 6 | 7 |
|  | While watching a movie in the cinema, two people beside Zhang are talking all the time. | 1 | 2 | 3 | 4 | 5 | 6 | 7 |
|  | Zhang sees that the colleague's hair is covered with dandruff, greasy and dirty. | 1 | 2 | 3 | 4 | 5 | 6 | 7 |
|  | Zhang squats and stands up. | 1 | 2 | 3 | 4 | 5 | 6 | 7 |
|  | Zhang is watching a comedy show. | 1 | 2 | 3 | 4 | 5 | 6 | 7 |
|  | The police catch Zhang stealing. | 1 | 2 | 3 | 4 | 5 | 6 | 7 |
|  | Zhang makes an appointment with the colleague at noon, but the colleague arrives two hours late. | 1 | 2 | 3 | 4 | 5 | 6 | 7 |
|  | Zhang takes the bread from the refrigerator and finds it moldy and covered with mould. | 1 | 2 | 3 | 4 | 5 | 6 | 7 |
|  | Zhang is standing on the roadside when suddenly a large group of children runs past him /her. | 1 | 2 | 3 | 4 | 5 | 6 | 7 |
|  | Zhang fails to pass the company's year-end review, so he/she can’t get promoted. | 1 | 2 | 3 | 4 | 5 | 6 | 7 |
|  | Zhang is surrounded by a pack of wolves. | 1 | 2 | 3 | 4 | 5 | 6 | 7 |
|  | Zhang learns that his/her best friend has leukemia. | 1 | 2 | 3 | 4 | 5 | 6 | 7 |
|  | Zhang tidies up the collar when he/she gets dressed. | 1 | 2 | 3 | 4 | 5 | 6 | 7 |
|  | The garbage can in Zhang's community emits a stench. | 1 | 2 | 3 | 4 | 5 | 6 | 7 |
|  | Zhang's bike seat is stolen. | 1 | 2 | 3 | 4 | 5 | 6 | 7 |
|  | Zhang drops the new iPhone into the water accidentally. | 1 | 2 | 3 | 4 | 5 | 6 | 7 |
|  | Zhang bites the apple and finds there is still half a worm in it. | 1 | 2 | 3 | 4 | 5 | 6 | 7 |
|  | Zhang is fired because of his/her gross negligence. | 1 | 2 | 3 | 4 | 5 | 6 | 7 |
|  | Zhang is about to travel around the world soon. | 1 | 2 | 3 | 4 | 5 | 6 | 7 |
|  | Zhang is taking the key to open the door. | 1 | 2 | 3 | 4 | 5 | 6 | 7 |
|  | Someone Zhang has a secret crush on politely refuses him/her. | 1 | 2 | 3 | 4 | 5 | 6 | 7 |
|  | Zhang is splashed with water by the speeding car. | 1 | 2 | 3 | 4 | 5 | 6 | 7 |
|  | Zhang is saying goodbye to boyfriend/girlfriend, and they will start exotic love about two years. | 1 | 2 | 3 | 4 | 5 | 6 | 7 |
|  | Zhang finds that the ugly girl ten years ago has become a beautiful woman. | 1 | 2 | 3 | 4 | 5 | 6 | 7 |
|  | Zhang does the same job as the colleague but only gets half the salary. | 1 | 2 | 3 | 4 | 5 | 6 | 7 |
|  | Zhang's score is only 3 points short of his/her favorite university. | 1 | 2 | 3 | 4 | 5 | 6 | 7 |
|  | Zhang sees a pig breaking into the classroom. | 1 | 2 | 3 | 4 | 5 | 6 | 7 |
|  | Zhang is listening to a lecture when he/she sees the teacher suddenly jumps onto the platform. | 1 | 2 | 3 | 4 | 5 | 6 | 7 |
|  | Zhang picks up the glass and drinks water. | 1 | 2 | 3 | 4 | 5 | 6 | 7 |
|  | The person that Zhang loves secretly confesses his/her love to Zhang actively. | 1 | 2 | 3 | 4 | 5 | 6 | 7 |

**Thanks for your participation again.**
