# Supplementary material for: Kinematic dataset of actors expressing emotions
Source: Sci Data. 2020 Sep 8;7:292. doi: 10.1038/s41597-020-00635-7 (PMC7478954; doi:10.1038/s41597-020-00635-7)
Supplement: Supplementary file 3 [file 41597_2020_635_MOESM3_ESM.docx]

**Instructions used in the recording phase**

1. **Free performance**
   1. Happiness

The next emotion you're going to perform is "happiness".

Before the performance, please imagine an event or scene that can make you "happy" and **imagine how you would react if you were in the situation.** If you're ready, tell us please. After we say “start”, you **exaggerate slightly** your reaction to the event or situation you've just associated that made you "happy". After the performance, tell us what you just associated with the event or scene.

Note: Your facial expression and body movement should be able to fully express your "happiness".

- 1. Anger

The next emotion you're going to perform is "anger".

Before the performance, please imagine an event or scene that can make you "angry" and **imagine how you would react if you were in the situation.** If you're ready, tell us please. After we say “start”, you **exaggerate slightly** your reaction to the event or situation you've just associated that made you "angry". After the performance, tell us what you just associated with the event or scene.

Note: Your facial expression and body movement should be able to fully express your "anger".

- 1. Sadness

The next emotion you're going to perform is "sadness".

Before the performance, please imagine an event or scene that can make you "sad" and **imagine how you would react if you were in the situation.** If you're ready, tell us please. After we say “start”, you **exaggerate slightly** your reaction to the event or situation you've just associated that made you "sad". After the performance, tell us what you just associated with the event or scene.

Note: Your facial expression and body movement should be able to fully express your "sadness".

- 1. Fear

The next emotion you're going to perform is "fear".

Before the performance, please imagine an event or scene that can make you "fearful" and **imagine how you would react if you were in the situation.** If you're ready, tell us please. After we say “start”, you **exaggerate slightly** your reaction to the event or situation you've just associated that made you "fearful". After the performance, tell us what you just associated with the event or scene.

Note: Your facial expression and body movement should be able to fully express your "fear".

1.5 Disgust

The next emotion you're going to perform is "disgust".

Before the performance, please imagine an event or scene that can make you "disgust" and **imagine how you would react if you were in the situation.** If you're ready, tell us please. After we say “start”, you **exaggerate slightly** your reaction to the event or situation you've just associated that made you "disgust". After the performance, tell us what you just associated with the event or scene.

Note: Your facial expression and body movement should be able to fully express your "disgust".

1.6 Surprise

The next emotion you're going to perform is "surprise".

Before the performance, please imagine an event or scene that can make you "surprise" and **imagine how you would react if you were in the situation.** If you're ready, tell us please. After we say “start”, you **exaggerate slightly** your reaction to the event or situation you've just associated that made you "disgust". After the performance, tell us what you just associated with the event or scene.

Note: Your facial expression and body movement should be able to fully express your "surprise".

1. **Scenario performance**

2.1 Happiness

The next emotion you're going to perform is "happiness".

Before the performance, we will read a script about "happiness", please listen carefully, **put yourself into the script situation, and imagine how you would react if you were in the situation.** If you're ready, tell us please. After we say "start", you'll get the reaction to the script.

Note: Your facial expression and body movement should be able to fully and **exaggerate slightly your "happiness".**

2.2 Anger

The next emotion you're going to perform is "anger".

Before the performance, we will read a script about "anger", please listen carefully, **put yourself into the script situation, and imagine how you would react if you were in the situation.** If you're ready, tell us please. After we say "start", you'll get the reaction to the script.

Note: Your facial expression and body movement should be able to fully and **exaggerate slightly your "anger".**

2.3 Sadness

The next emotion you're going to perform is "sadness".

Before the performance, we will read a script about "sadness", please listen carefully, **put yourself into the script situation, and imagine how you would react if you were in the situation.** If you're ready, tell us please. After we say "start", you'll get the reaction to the script.

Note: Your facial expression and body movement should be able to fully and **exaggerate slightly your "sadness".**

2.4 Fear

The next emotion you're going to perform is "fear".

Before the performance, we will read a script about "fear", please listen carefully, **put yourself into the script situation, and imagine how you would react if you were in the situation.** If you're ready, tell us please. After we say "start", you'll get the reaction to the script.

Note: Your facial expression and body movement should be able to fully and **exaggerate slightly your "fear".**

2.5 Disgust

The next emotion you're going to perform is "disgust".

Before the performance, we will read a script about "disgust", please listen carefully, **put yourself into the script situation, and imagine how you would react if you were in the situation.** If you're ready, tell us please. After we say "start", you'll get the reaction to the script.

Note: Your facial expression and body movement should be able to fully and **exaggerate slightly your "disgust".**

2.6 Surprise

The next emotion you're going to perform is "surprise".

Before the performance, we will read a script about "surprise", please listen carefully, **put yourself into the script situation, and imagine how you would react if you were in the situation.** If you're ready, tell us please. After we say "start", you'll get the reaction to the script.

Note: Your facial expression and body movement should be able to fully and **exaggerate slightly your "surprise".**

2.7 Neutral

The next emotion you're going to perform is "neutral".

Before the performance, we will read a script about "neutral", please listen carefully, **put yourself into the script situation, and imagine how you would react if you were in the situation.** If you're ready, tell us please. After we say "start", you'll get the reaction to the script.

Note: Your facial expression and body movement should be able to fully and **exaggerate slightly your "neutral".**
